# Supplementary material for: Strong graphene oxide nanocomposites from aqueous hybrid liquid crystals
Source: Nat Commun. 2020 Feb 11;11:830. doi: 10.1038/s41467-020-14618-0 (PMC7012915; doi:10.1038/s41467-020-14618-0)
Supplement: Supplementary file 1 — Supplementary Information [file 41467_2020_14618_MOESM1_ESM.pdf]

Supplementary Information for

Strong Graphene Oxide Nanocomposites from Aqueous Hybrid Liquid Crystals

Hegde et al

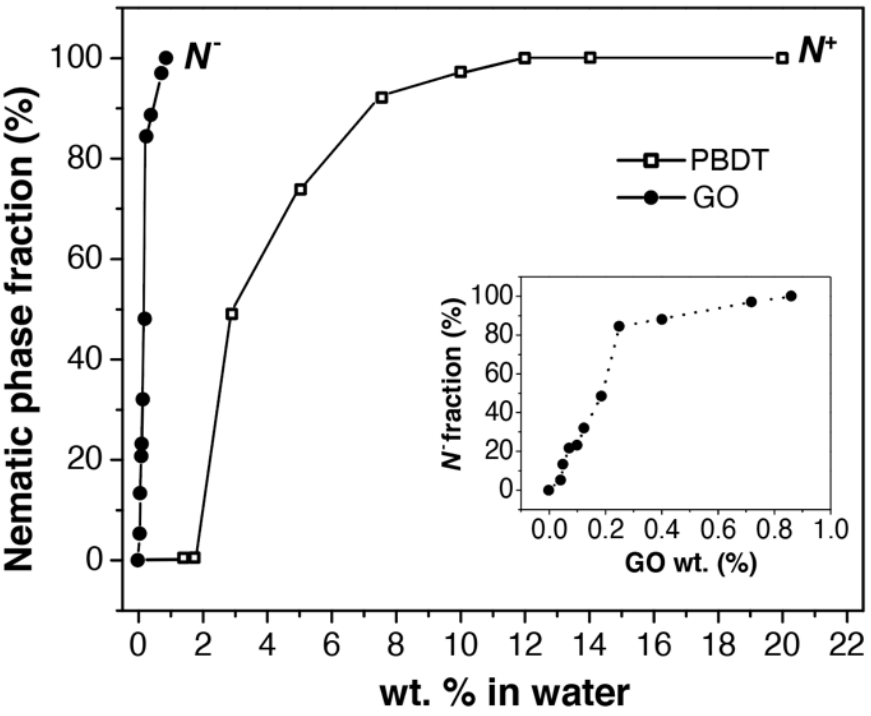

Supplementary Figure 1. **Nematic phase fraction of PBDT solutions and colloidal GO platelets.** PBDT (denoted by open squares) is isotropic below 1.9 wt.% and is fully nematic above 12.0 wt.%. GO colloids (denoted by black circles) are isotropic below 0.018 wt.% and become fully nematic above 0.9 wt.%. *Inset*: Nematic phase fraction as a function of GO concentration is shown on an expanded scale for clarity. Source data are provided as a Source Data file.

Supplementary Note 1: Based on the I-N transition for PBDT, the axial rigidity persistence length (L_p) of PBDT can be calculated using Onsager theory (equation 1)[1,2,3].

$$\varphi_{\text{nematic}} \cong 4.5\ D/L_p \qquad (1)$$

In the above equation, D denotes the bundle diameter (~ 0.8 nm)[2] and φ_{nematic} is the volume fraction of rods at which the nematic phase is formed. The *I-N* transition occurs at 1.9 wt.% i.e. φ_{nematic} = 0.015 volume fraction assuming a polymer density of 1.4 g·cm^{-3}.

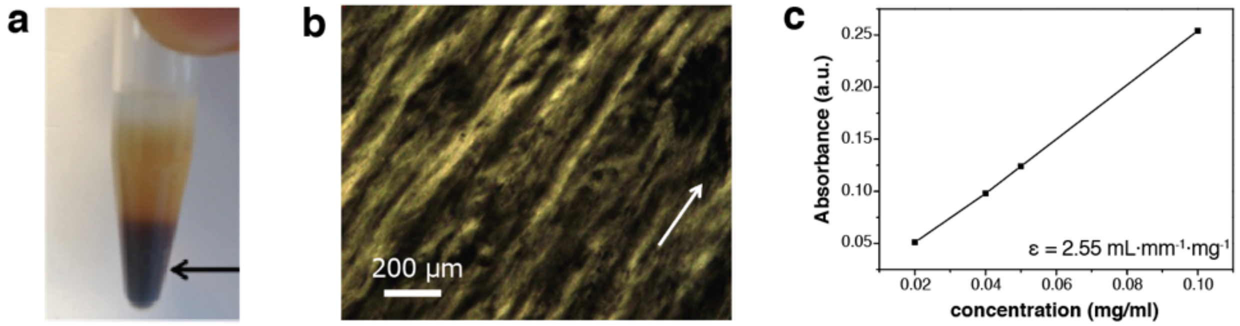

Supplementary Figure 2. GO size selection, liquid crystallinity, and concentration measurements. (**a**) The washing steps, mild centrifugation at 500 RPM, low-intensity sonication followed by dialysis yields an isotropic GO colloid. Centrifugation at 3500 RPM causes this solution to fractionate; a top isotropic fraction with extremely low GO concentration, middle isotropic fraction that contains small (low-aspect ratio) GO flakes and a dark, liquid crystalline fraction at the bottom (dark layer marked with an arrow). The bottom layer was collected from all the centrifuged tubes and used for preparing nanocomposites. (**b**) The dark, isolated fraction was transferred to a Vitrotube capillary (I.D. = 0.2 mm) by capillary action and imaged between crossed polarizers of an optical microscope. The liquid crystalline phase (nematic, N^-) results in birefringent textures between crossed polarizers of an optical microscope. The arrow indicates the shear direction. Scale bar in is 200 µm. (c) The concentration of liquid crystalline GO colloids was measured using UV-Vis and corroborated using scanning thermo-gravimetric analysis (not shown here). The extinction coefficient was determined to be 2.5 $mL\cdot mm^{-1}\cdot mg^{-1}$. The concentration of liquid crystalline colloidal GO from centrifugation was found to be 2.5 $mg\cdot mL^{-1.}$ Source data are provided as a Source Data file.

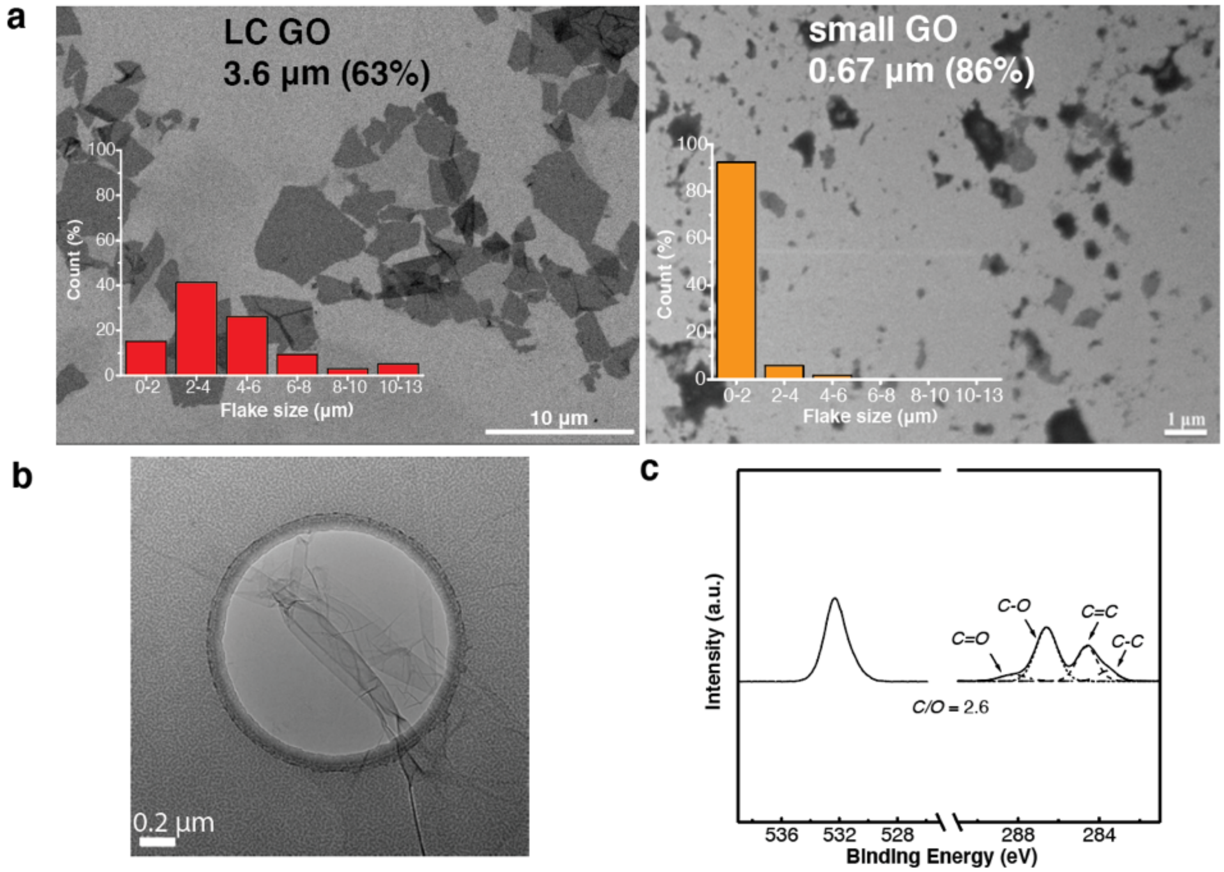

Supplementary Figure 3. **Characterization of GO flakes; size and C/O ratio** (**a**) Liquid crystalline GO flakes obtained from centrifugation were deposited on a Si wafer as described in the experimental section and visualized using SEM. Statistical GO flake size analysis of ~120 GO flakes yields an average lateral flake size of 3.64 μm with a polydispersity of 63%. Flake size is defined as the longest span and was measured using ImageJ software. The polydispersity (%) is defined as relative standard deviation.[4] Low-intensity bath sonication of 1 mL liquid crystalline GO for 1 h causes GO flake scission and reduces the average flake size to 0.67 μm with a polydispersity of 86%. The resulting solution is isotropic. (**b**) TEM image of liquid crystalline GO flakes deposited on a holey grid. The GO is transparent and no dark patches due to impurities are visible. By counting the number of parallel lines from GO flake edges, the number of stacked GO layers can be estimated. Although curling (evident in this image) can obfuscate the edge counting method, a majority of the deposited GO flakes were either monolayer or bilayer. The layer number obtained from any visualization technique (TEM, SEM or AFM) is often biased towards lower layer numbers as highly diluted solutions are often utilized for deposition. However, there is enough evidence from previously published scattering studies to support the existence of mostly

monolayer GO in liquid crystal phases of colloidal GO.[5] (**c**) X-ray photoelectron spectroscopy (XPS) of as-synthesized GO. The *C/O* ratio for GO was found to be 2.6. Source data are provided as a Source Data file.

Supplementary Table 1: Component (PBDT and GO) concentrations in the hybrid mixtures.

C_{total} [a] (wt.%)	C_P [b] (wt.%)	C_{GO} [b] (wt.%)	Mass fraction, F_{GO} GO/(GO+PBDT)
1.7 (*I*)	1.67 (*I*)	0.041 (I+N^-)	0.0244
2.8 (I+^hN_b)	2.75 (I+N^+)	0.069 (I+N^-)	0.0244
4.9 (^hN_b)	4.75 (I+N^+)	0.120 (I+N^-)	0.0244
7.1 (^hN_b)	6.89 (I+N^+)	0.173 (I+N^-)	0.0244
14.4 (^hN_b)	14.04 (N^+)	0.351 (I+N^-)	0.0244

[a] The phase of hybrid composite mixtures are reported in parenthesis. [b] The phase reported in parenthesis refers to that of the individual components *i.e.* PBDT solution or colloidal GO, from fig. S1. ***Abbreviations***: C_{total} = total solids concentration = (PBDT+GO)/(PBDT+GO+H_2O); expressed in wt.%, C_P = concentration of polymer in hybrid mixture, C_{GO} = concentration of GO in hybrid mixture, N^+ = nematic phase formed by PBDT rods, N^- = mesoscopic nematic phase formed by GO platelets, I = isotropic, ^hN_b.= hybrid biaxial nematic.

Supplementary Table 2: Parameters relevant to hybrid nematic phase formation in PBDT+GO precursor mixtures[a]

C_{total} (wt.%)	C_P (wt.%)	C_{GO} (wt.%)	Density of precursor mixture (*d*) g/cm³	$Ø_{GO}$	V_{eff} µm³	H µm
1.7 (*I*)	1.67	0.041	1.005	0.00021	39.56	3.9
2.8 (I+^hN_b)	2.75	0.069	1.008	**0.00035**	**23.43**	**2.3**
4.9 (^hN_b)	4.75	0.12	1.014	**0.00061**	**13.39**	**1.3**
7.1 (^hN_b)	6.89	0.173	1.021	**0.00088**	**9.23**	**0.9**
14.4 (^hN_b)	14.04	0.351	1.044	**0.00183**	**4.45**	**0.4**

[a]The method used for calculating *d*, $Ø_{GO}$, V_{eff}, and *H* are shown below under Calculation methodology. ***Abbreviations***: $Ø_{GO}$= volume fraction of graphene oxide, V_{eff} =effective volume per GO platelet, *H*= interplatelet distance.

Calculation methodology:

The volume of a single GO platelet = $V_{GO} = \pi r^2 t$ = 0.00815 μm^3 (here r = radius of GO platelet = 1.8 μm, t = thickness = 0.8 nm[6]). The expression $\frac{1}{d} = \sum \frac{C_i}{d_i}$ yields the density (d) of the PBDT+GO mixture; d_{GO} is ~2.0 g/cm^3 (ref. 6 in S.I) and C_i and d_i are mass concentrations (wt. %) and densities of individual components (GO, PBDT and water). The volume fraction of graphene oxide ($\emptyset_{GO}$) in the mixture can be calculated from $\emptyset_{GO} = \frac{d}{d_{GO}} C_{GO}$. The number density ρ_{NGO} can be estimated using the expression $\rho_{NGO} = \frac{\emptyset_{GO}}{V_{GO}}$. The effective volume per GO platelet (V_{eff}), i.e., the accessible volume per GO platelet in the mixture, can be calculated using the equation $V_{eff} = \frac{1}{n_{GO}}$.

According to Onsager[1], the excluded volume, $V_{excl,\,GO}$ of randomly oriented GO platelets with thickness t and diameter D, is given by:

$$V_{\text{excl,GO}} = \frac{1}{4}\pi D \left(L^2 + \frac{1}{2}(\pi + 3)tD + \frac{1}{4}tD^2 \right)$$

Since $t \ll D$, this can be approximated to $V_{\text{excl,GO}} = \frac{\pi^2}{16} D^3 \sim 29$ μm^3 (D = diameter of GO = 3.6 μm). We can estimate the inter-platelet distance using the expression[7,8] : $H = \frac{t}{\emptyset_{GO}}$. Additionally, the critical overlap concentration for GO ($\emptyset^*_{GO}$) can also be calculated using the following expression:

$$\emptyset^*_{GO} = \frac{V_{\text{disk}}}{V_{\text{swept sphere}}} = \frac{3}{4}\frac{t}{r} = 0.00033$$

From Supplementary Table 2, it is evident that $\emptyset_{GO}$ exceeds $\emptyset^*_{GO}$ for $C_{total} \geq 2.8$ wt.%. Additionally, V_{eff}, H are lower than $V_{excl,GO}$ and D, indicating significant overlap of GO platelets.

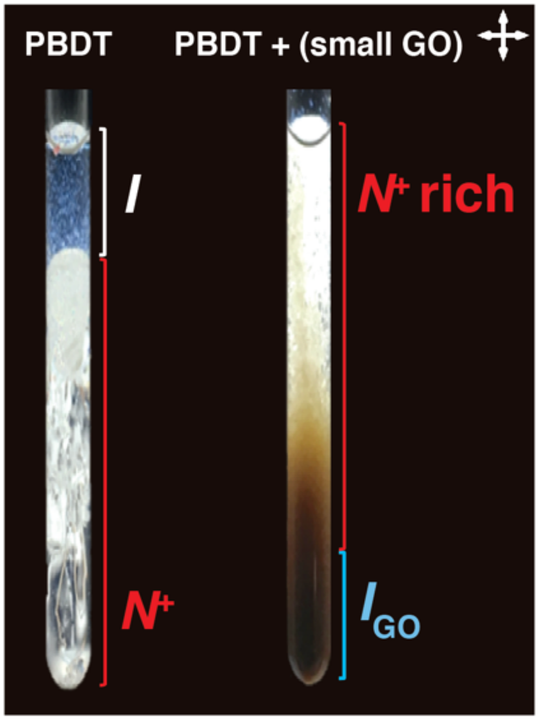

Supplementary Figure 4. **Phase behavior of PBDT+small GO composite mixture.** At C_P = 4.75 wt.%, PBDT is biphasic and mild centrifugation at 600 RPM for 30 min. causes phase separation into two phases; *I* (27 %) and N^+ (73 %). Centrifugation of PBDT+ small-GO hybrid mixture (F_{GO} = 0.0244) at C_{total} = 4.9 wt.% results in phase separation into PBDT-rich upper nematic and a lower isotropic phase containing PBDT with aggregated GO. The phase inversion, *i.e.* upper nematic and lower isotropic, in PBDT+(small GO) relative to pure PBDT is due to sedimentation of dense, flocculated isotropic GO. The flocculated GO tends to strongly adhere to the glass wall, but measurement of the nematic and isotropic layers after isolation yields phase fraction values similar to neat PBDT (left tube).

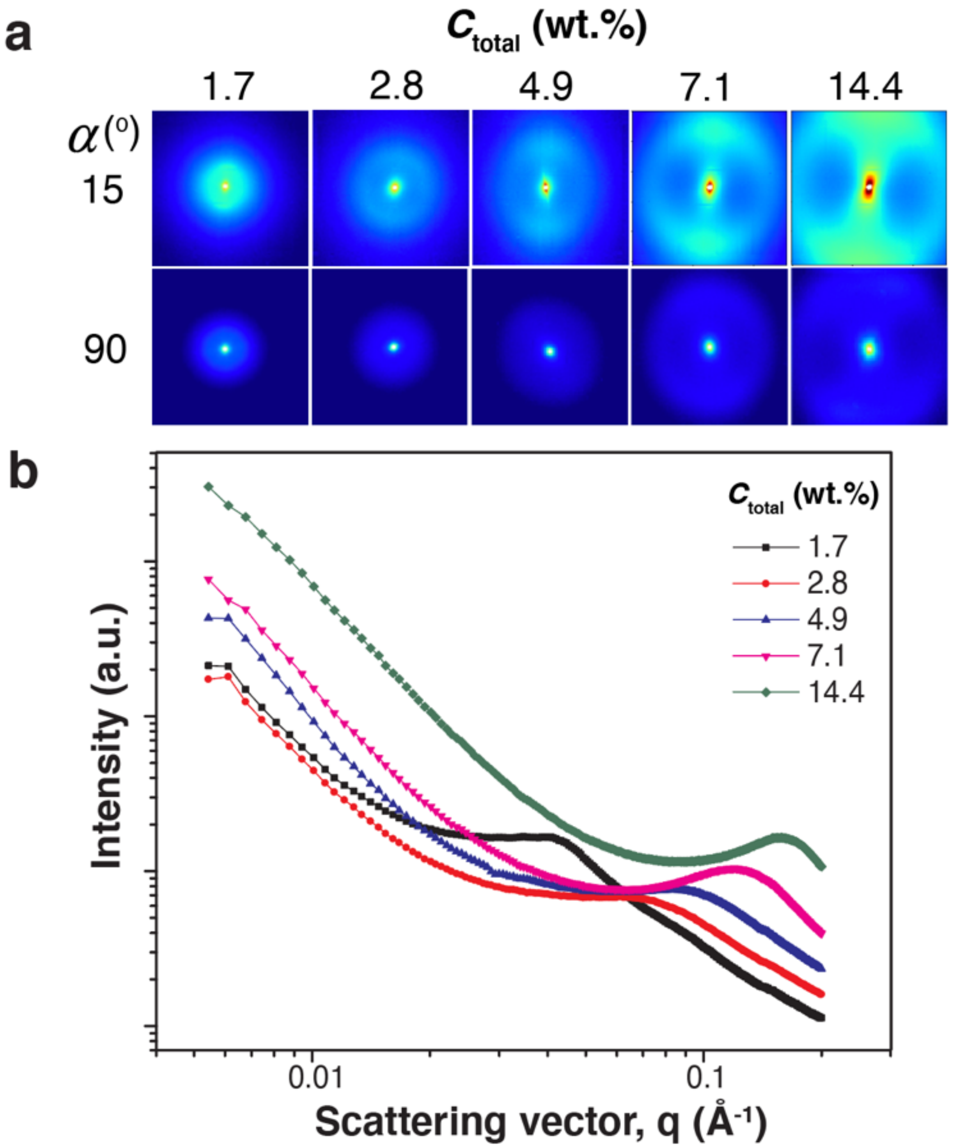

Supplementary Figure 5. Transmission small-angle scattering from PBDT+GO hybrid mixtures. (a) Scattering from hybrid mixtures at $\alpha \approx 15^o$ (edge-on) and at 90° (normal incident). The homogenous texture of PBDT and homeotropic alignment of GO in the PBDT+GO mixture results in isotropic scattering patterns when measured at $\alpha = 90^o$ (normal incident). At $C_{total} = 7.1$ wt.%, the hybrid mixture exhibits weak, local anisotropy even at $\alpha = 90^o$ due to shear-induced orientation introduced during sample loading. The anisotropy of hybrid nematic composites (above $C_{total} = 4.9$ wt.%) is clear when measured at $\alpha \approx 15^o$ (edge-on). (**b**) Average intensity as a function of scattering vector **q** from a radial cut of the SAXS scatting patterns (radial vector lies along the GO and PBDT scattering) obtained at $\alpha \approx 15^o$ for all hybrid composite mixtures. The scattering

intensities for different C_{total} values in Supplementary Fig. 5b are reported in arbitrary units and are not directly comparable—measurements were performed on different samples with minor experimental variations. Legend: Filled black square denotes 1.7 wt.%, filled red circle denotes 2.8%, blue triangle denotes 4.9 wt.%, pink triangle denotes 7.1 wt.%, green diamonds denotes 14.4 wt.%. Source data are provided as a Source Data file.

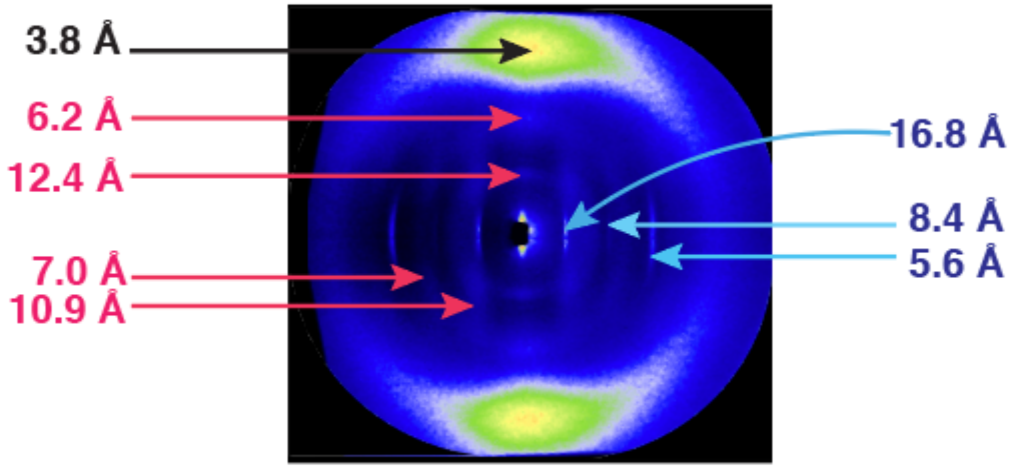

Supplementary Figure 6. WAXS analysis of neat PBDT film. WAXS scattering pattern from neat PBDT film measured at $\alpha = 0^{o}$ (edge-on). The spacings corresponding to the scattering peaks are indicated in the figure. The sharp equatorial reflections correspond to the characteristic repeat length of the monomeric unit (16.8 Å) and its first two harmonics (8.4 Å and 5.6 Å). The weak diffuse reflections associated with the spacings 6.2 Å, 7.0 Å, 10.9 Å and 12.4 Å arise from an underlying transverse 2D lattice of poorly ordered polymer chains (para-crystal). Finally, the broad, intense meridional diffuse at 3.8 Å corresponds to the average inter-rod distance of the amorphous polymer. The reflections corresponding to 3.8 Å, 5.6 Å, 8.4 Å, and 16.8 Å change into isotropic rings when the film is investigated at $\alpha = 90^{o}$ (normal incidence). The reflections that are marked in red are not observed when measured at normal incidence. All the reflections are also observed in PBDT+GO films although their intensity and azimuthal broadening are dependent on the C_{total} utilized for nanocomposite preparation.

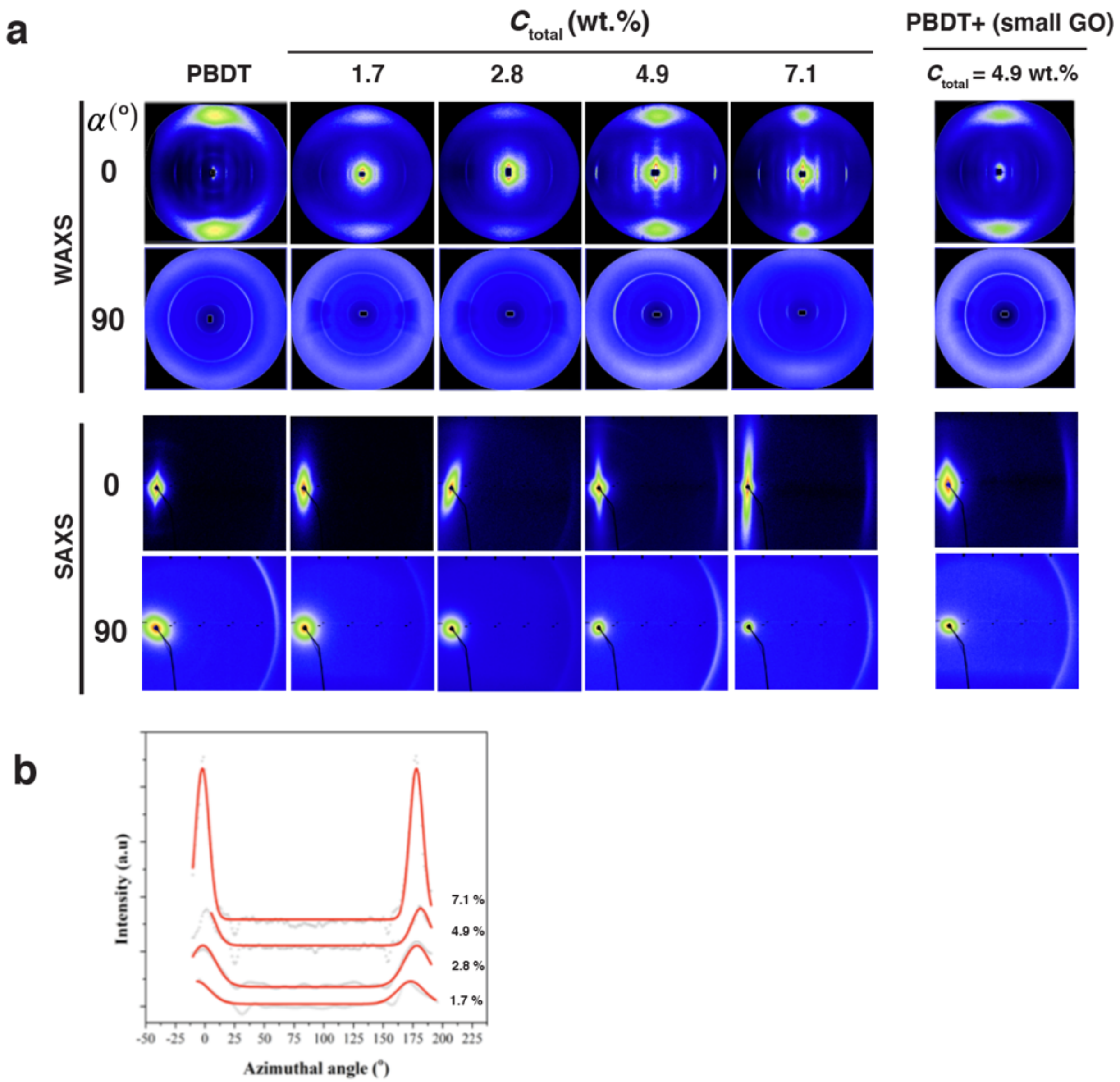

Supplementary Figure 7. WAXS and SAXS scattering patterns for PBDT and PBDT+GO nanocomposite films. (a) Scattering patterns of films cast from different C_{total} mixtures, measured at α = 0° (edge-on, horizontal film) and α = 90° (normal incidence). Scattering patterns for PBDT+(small GO) nanocomposite film (from C_{total} = 4.9 wt.%) are shown on the right. The metastable PBDT+(small GO) hybrid mixture results in nanocomposite films with a low $<P_2>$ order parameter of ≈ 0.5. **(b)** Maier-Saupe fits (red curves) to the azimuthal intensity profiles (gray dotted curve) from edge-on SAXS patterns along circles (highlighted in the first pattern) at **q** =

0.14 Å^{-1} (d = 45 Å). The total solids concentration of the solutions used to make the composite films are listed alongside the curves. Curves have been vertically translated for clarity.

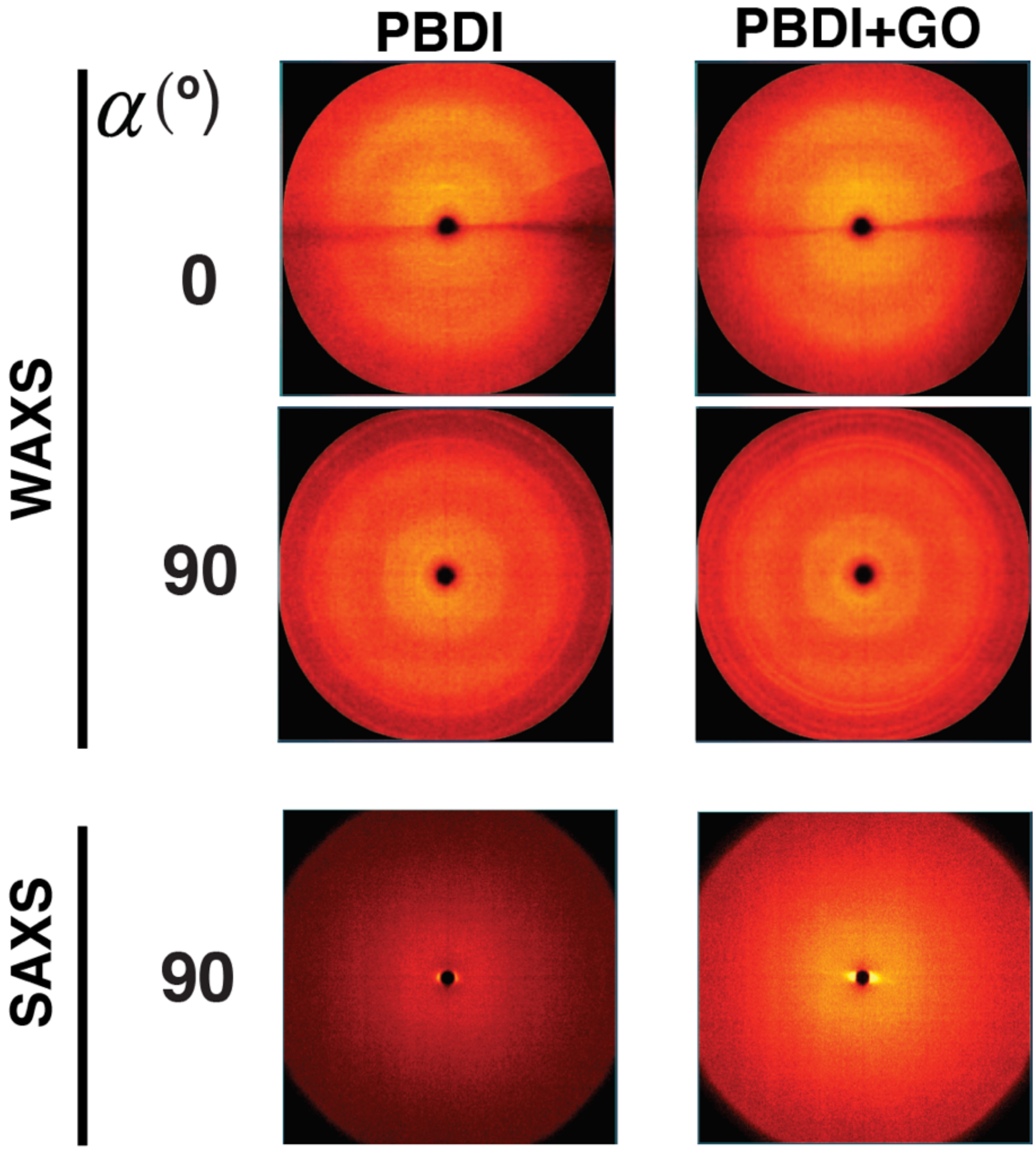

Supplementary Figure 8. WAXS and SAXS patterns of PBDI and PBDI+GO films. The measurements were taken with an in-house Bruker AXSD8 Discover diffractometer as described in the experimental section. PBDI solutions are isotropic and film casting and drying results in isotropic films *i.e.* the polymer chains have no preferential orientation along any axes. The

prominent scattering peaks are attributed to intramolecular repeats (14.8 Å from monomer repeat units and 6.8 Å from intramonomer sulfonate distance) and interchain distance (3.8 Å).[9] The inclusion of GO in PBDI does not result in noticeable changes in the WAXS and SAXS scattering patterns (either in anisotropy or intensity) regardless of the incidence angle.

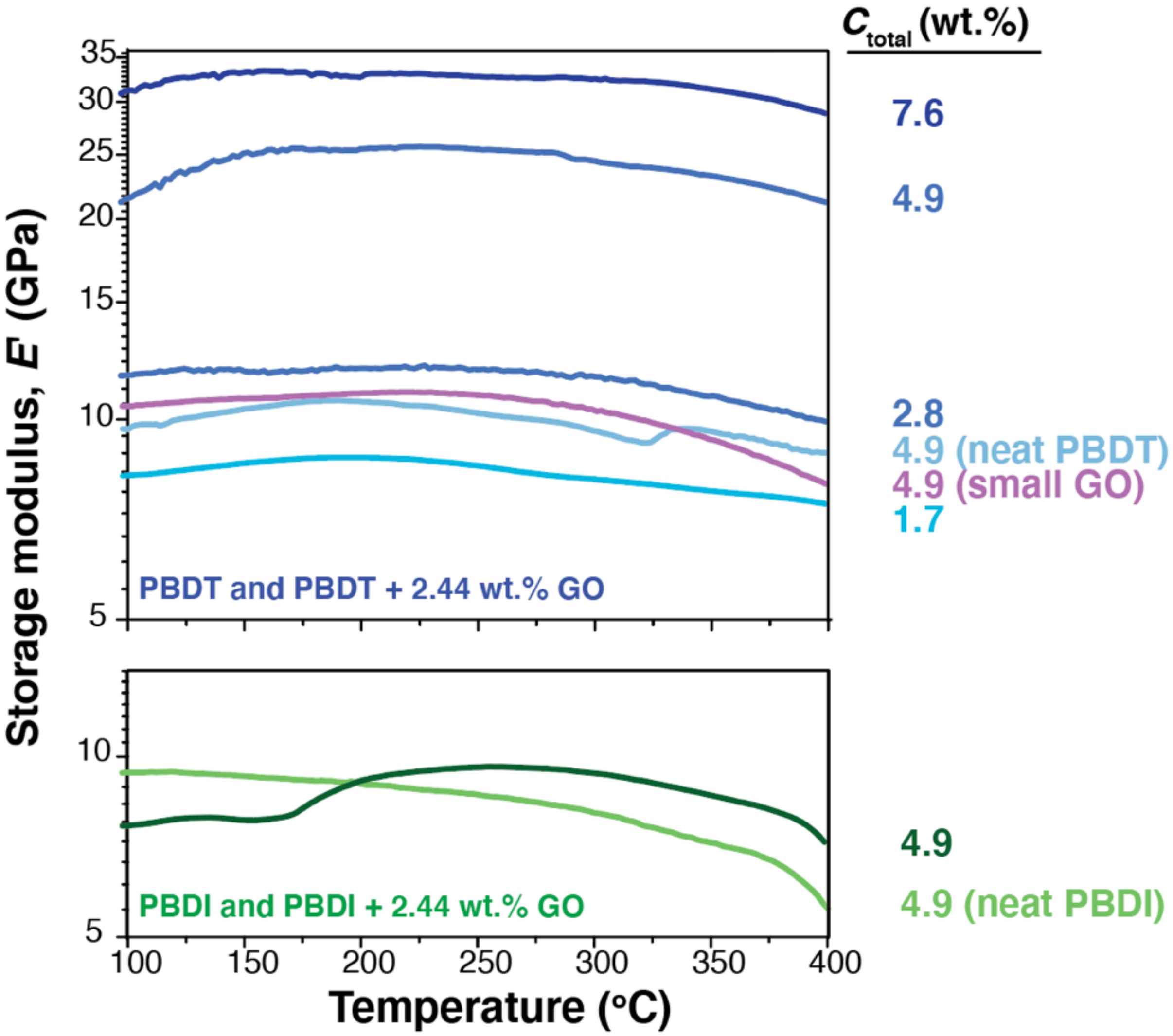

Supplementary Figure 9. **Dynamic mechanical thermal analysis (DMTA) of nanocomposite films.** Storage modulus (*E*') at 1 Hz as a function of temperature (2 °C/min heating rate). The C_{total} used for casting the films is listed on the right. The storage modulus values used in plotting modulus enhancement were obtained at 200 °C. As the films are hygroscopic, water evaporation causes storage modulus values to fluctuate (by 1-3 GPa) at temperatures lower than 150 °C. Source data are provided as a Source Data file.

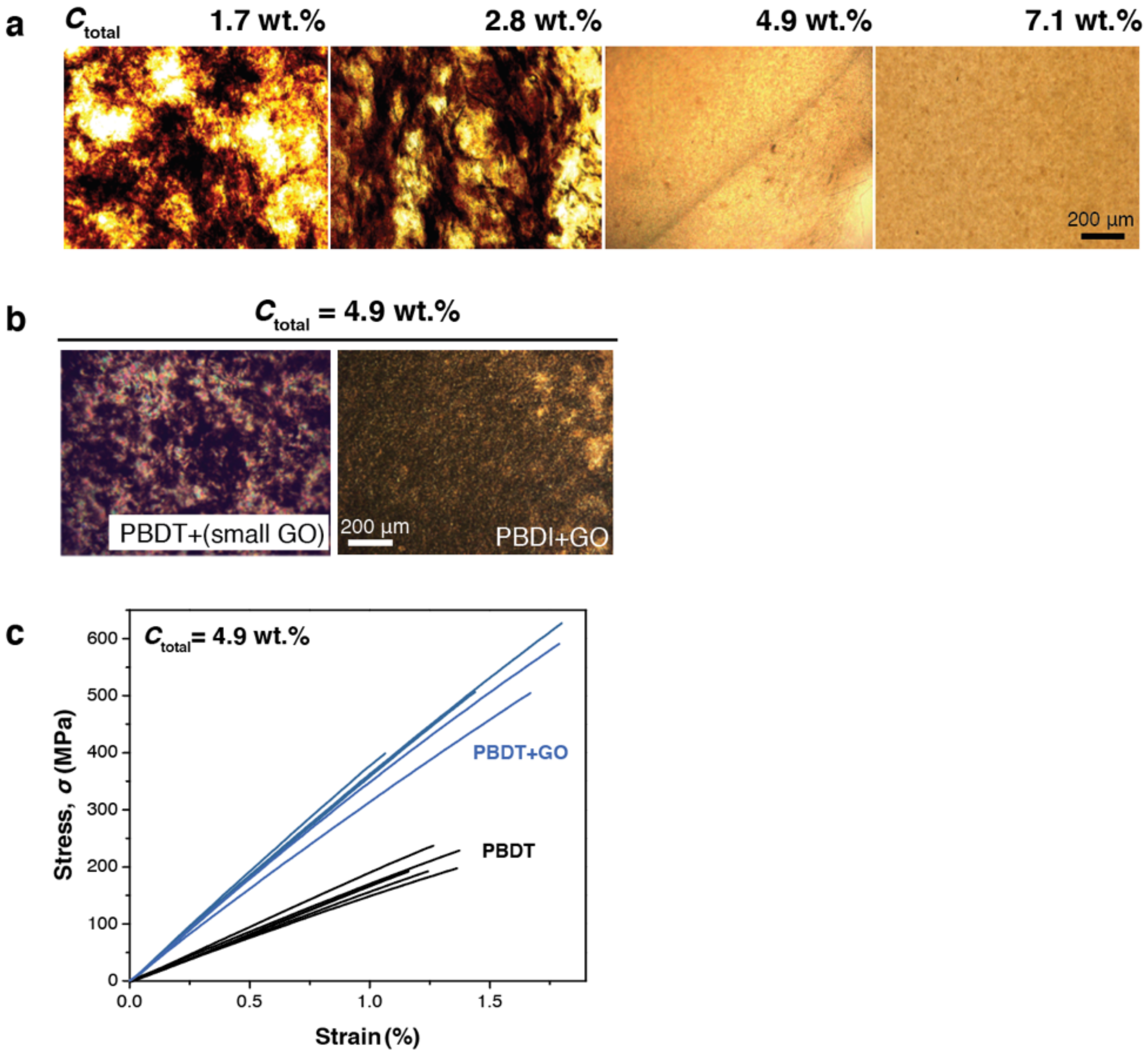

Supplementary Figure 10. Optical micrographs and stress-strain analysis of nanocomposite films. (**a**) The C_{total} used for preparing PBDT+GO films are listed above the optical micrographs. The scale bar for all micrographs is the same and is shown in the micrograph for C_{total} = 7.1 wt.%. Large scale GO aggregation is only visible in films cast from 1.7 and 2.8 wt.% hybrid mixtures. (**b**) Visible GO aggregation in the optical micrographs for PBDT+(small GO) and PBDI+GO nanocomposites cast from unstable C_{total} = 4.9 wt.% hybrid mixtures. (**c**) Tensile stress strain curves for PBDT and PBDT+GO nanocomposite films. A total of 5 measurements were performed for each sample type. PBDT+ GO nanocomposite films (C_{total} = 4.9 wt.%) exhibit improved mechanical properties: The Young's modulus increases from 16 (±1) GPa to 36 (±2) GPa, and the

average tensile strength increases from ~210 (±20) MPa to ~525 (±90) MPa. The values in parenthesis are standard deviations from the average of at least five measurements. Source data are provided as a Source Data file.

Supplementary Table 3: Mechanical properties of PBDT and PBDT+GO nanocomposite films

PBDT				PBDT+GO		
Test no.	Modulus GPa	Strength Mpa	Strain %	Modulus GPa	Strength Mpa	Strain %
1	15.2	193	1.16	34.8	591	1.79
2	18.6	237	1.26	37.3	627	1.80
3	15.1	192	1.24	37.9	399	1.06
4	15.9	192	1.16	35.3	506	1.44
5	17.3	229	1.37	33.0	505	1.67
6	15.2	197	1.36			
Average	16.2	207	1.26	35.7	525	1.55

Supplementary References:

1 Onsager, L. The effects of shape on the interaction of colloidal particles. *Ann. NY. Acad. Sci.* **51**, 627-659 (1949).

2 Wang, Y., He Yadong, Yu, Z., Gao, J., ten Brinck, S., Slebodnick, C., Fahs, G. B., Zanelotti, C. J., Hegde, M., Moore, R. B., Ensing, B., Dingemans, T. J., Qiao, R., Madsen, L. A. Double helical conformation and extreme rigidity in a rodlike polyelectrolyte. *Nat. Commun.*, **10,** 801:1-8 (2019).

3 Viale, S., Best, A. S., Mendes, E., Jager, W. F., Picken, S. J. A supramolecular nematic phase in sulfonated polyaramides. *Chem. Commun.*, 1596-1597 (2004).

4 van der Kooij, F. M., Lekkerkerker, H. N. W. Liquid-crystal phases formed in mixed suspensions of rod- and platelike colloids. *Langmuir* **16**, 10144-10149 (2000).

5 Poulin, P., Jalili, R., Neri, W., Nallet, F., Divoux, T., Collin, A., Aboutalebi, S. H., Wallace, G., Zakri, C. Superflexibility of graphene oxide. *PNAS* **113**, 11088-11093 (2016).

6 Tkacz, R., Abedin, Md J., Sheath, P., Mehta, S. B., Verma, A., Oldenbourg, R., Majumder, M., Phase Transition and Liquid Crystalline Organization of Colloidal Graphene Oxide as a Function of pH, *Part. Part. Syst. Charact.*, **34**, 1600391:1-11 (2017).
7 Gabriel, J. C. P., Camerel, F., Lemaire, B. J., Desvaux, H., Davidson, P., Batail, P. Swollen liquid-crystalline lamellar phase based on extended solid-like sheets. *Nature*, **413**, 504-508 (2001).
8 Paineau, E., Michot, L. J., Bihanicc, I., Baravian, C. Aqueous Suspensions of Natural Swelling Clay Minerals. 2. Rheological Characterization. *Langmuir*, **27**, 7806-7819 (2011).
