# Supplementary material for: Strong graphene oxide nanocomposites from aqueous hybrid liquid crystals
Source: Nat Commun. 2020 Feb 11;11:830. doi: 10.1038/s41467-020-14618-0 (PMC7012915; doi:10.1038/s41467-020-14618-0)
Supplement: Supplementary file 3 — Description of Additional Supplementary Files [file 41467_2020_14618_MOESM3_ESM.pdf]

Description of Additional Supplementary Files

File Name: Supplementary Data 1

Description: The enhancement in modulus (ΔE) and tensile strength (Δσ) for polymer+GO nanocomposite films.
