# Supplementary material for: Strong graphene oxide nanocomposites from aqueous hybrid liquid crystals
Source: Nat Commun. 2020 Feb 11;11:830. doi: 10.1038/s41467-020-14618-0 (PMC7012915; doi:10.1038/s41467-020-14618-0)
Supplement: Supplementary file 4 — Supplementary Data 1 [file 41467_2020_14618_MOESM4_ESM.pdf]

Supplementary Data 1 for

Strong Graphene Oxide Nanocomposites from Aqueous Hybrid Liquid Crystals

Hegde et al

Supplementary Data 1. The enhancement in modulus (ΔE) and tensile strength (Δσ) for polymer+GO nanocomposite films.[a]

No.	Matrix	GO type[a]	Test[b]	GO [c] wt.%	E_{PM} GPa	ΔE GPa	σ_{PM} MPa	Δσ[d] MPa	Reference
**Aliphatic polymers**									
1	Polyethylene succinate	GO	Tensile	0.5	0.38	0.03	38	16	1
2	HDPE/MAPE (90/10)	GO	Tensile	0.3	0.43	0.15	11.6	1.4	2
3	Polypropylene (PP)	rGO	Tensile	10	0.98	0.52	NA	NA	3
4	polyethylene oxide (PEO)	GO	Tensile	0.5	0.3	2.9	38	14	4
5	Nafion	GO	Tensile	4.5	NA	NA	9.4	70.07	5
6	Poly (lactic acid) (PLLA)	GOf	Tensile	1	1	0	33	42	6
7	PLLA	GO	Tensile	1	1	0	33	17	7
8	PLLA	GO	Tensile	2	1.56	0.93	41.4	21.6	2
9	Polycaprolactone (PCL)	GO	Tensile	2	0.21	0.23	14.2	13.3	2
10	PCL	GO	Tensile	2	0.25	0.065	NA	NA	7
11	PCL	rGO	Tensile	2	0.25	0.165	NA	NA	7
12	chitosan-PVP	GO	Tensile	0.5	2	1.5	60	60	8
13	Polyester resin +MEKP hardner	rGO	Tensile	3	1.6	1.4	26	32	9
14	Polyester-Carbon fiber	rGO	DMTA	10.3	0.5	0.5	NA	NA	10
15	Polyester-Carbon fiber	rGO	Tensile	10.3	NA	NA	1.75	0.65	10
16	Polyester	GO	Tensile	3	1.2	0.5	35	40	9
17	Polyamide 6	TrGO	Tensile	10	1.65	0.78	NA	NA	3
18	Polycarbonate (PC)	TrGO	Tensile	10	1.48	1.19	NA	NA	3
19	PC	rGO	Tensile	1	1.9	0.9	62	26	11
20	Linear PC	GOf	Tensile	3	0.09	0	NA	NA	12
21	Branched PC	GOf	Tensile	0.5	0.072	-0.012	NA	NA	12
22	PC	rGOf	Tensile	3	1.9	0.2	51	3	13
23	Polyvinyl alcohol (PVA)	GO	Tensile	5.4	2.9	3	33.00	29	14
24	PVA	GO	Tensile	5.4	2.5	1.5	64.00	3	14
25	PVA	GO	Tensile	0.5	2.1	1.2	41.5	11.9	15
26	PVA	GOf	Tensile	1.4	0.16	0.37	22.5	23.2	16

27	PVA	GOf	Tensile	0.525	2.75	1.35	90	50	17
28	PVA	GO	Tensile	1	3.8	3.4	90	40	18
29	PVA	GO	Tensile	5	2.1	4.0	63	22	19
30	PVA	GO	Tensile	20	-	-	16	43.6	20
31	PVA	rGO	Tensile	1	4.1	1.4	97	41	21
32	PVA	GO	Tensile	1	3.75	3.75	95	40	22
33	PVA	rGO	Tensile	0.3	2.32	0.23	25.3	13.7	23
34	PVA	GO	Tensile	0.3	2.32	3.5	25.3	37.7	22
35	PVA	GO	Tensile	0.5	0.45	0.03	22	3	24
36	PVA	rGO	Tensile	4.97	0.1	1	15	28	25
37	chitosan-PVA	GO	Tensile	6	1.83	3.95	52	13	26
38	Polymethylmethcrylate (PMMA)	GOf	Tensile	1	0.0187	0.0031	35.5	6.5	27
39	PMMA	GO	Tensile	1	0.0187	0	35.5	-2.8	26
40	PMMA	GOf	Tensile	1	1.2	0.75	34	16	28
41	PMMA-HA bone cement	GO	DMTA	0.5	2.67	0.48	NA	NA	26
42	PMMA	GO	DMTA	1	2.5	0.5	NA	NA	29
43	PMMA	GO	Tensile	1	2.1	0.8	76.5	16.065	28
44	PMMA-PEO	GO	DMTA	6.7	0.52	0.619	NA	NA	30

Semi-aromatic polymers

45	Polystyrene	GO	Tensile	2	1.83	1.75	23	20.5	2
46	Polyurethane/Epoxy	GO	Tensile	0.033	0.218	0.079	17.2	2.2	31
47	Epoxy	GOf	Tensile	0.5	3.15	0.45	52.9	12.0	32
48	Epoxy	GO	Tensile	0.5	3.15	0.52	52.9	32.5	32
49	Epxoy	GOf	DMTA	0.5	1.841	0.22	NA	NA	32
50	Epxoy	GOf	Tensile	0.2	2.5	0.8	69	12	33
51	Epxoy	GOf	DMTA	0.4	1.5	1.7	NA	NA	34
52	Epxoy	GOf	Tensile	1	2.8	0.8	140	30	35
53	Polyethyleneterepthalate	GO	DMTA	7	2.2	1.2	NA	NA	36
54	PI (P84)	GOf	Tensile	0.2	2.03	0.71	77.40	20.6	28

Aromatic Polymers

55	Polyimide (PI) (sBPADA-BAPP)	GOf	DMTA	4	1	1.25	NA	NA	37
56	PI (BTDA-ODA-PA)	GOf	Tensile	0.75	1.81	1.12	128.00	76	38
57	PI (PMDA-ODA)	GOf	Tensile	0.3	2.3	0.8	86.00	22	39
58	PI (PMDA-ODA)	rGO	Tensile	0.3	2.3	0.05	NA	NA	41
59	PI (PMDA-ODA)	rGO	Tensile	2	2.5	3.1	75.70	-8.7	40
60	PI (PMDA-ODA)	GOf	Tensile	2	2.5	7.1	75.70	22.3	42

71	PI (PMDA-ODA)	rGOf	Tensile	0.75	1.8	0.6	122.00	5	41
72	PI (PMDA-ODA)	GO	Tensile	1	1.347	0.333	87.00	4	42
73	PI (PMDA-ODA)	GO	Tensile	1	2.215	0.455	73.80	6.2	44
74	PI (PMDA-ODA)	rGO	Tensile	0.5	1.8	0.2	88.00	32	43
75	PI (PMDA-ODA)	GO	Tensile	0.5	1.8	0.2	88.00	4	45
76	Polybenzimidazole	GO	Tensile	1	3.2	0.75	145	26	44
77	Poly ether ether ketone	GOf	Tensile	3	2.2	0.9	100	5	45
78	Polyarylene ether nitrile	GOf	DMTA	0.75	1.95	1.31	NA	NA	46
79	Polyarylene ether nitrile	GOf	Tensile	0.75	3.26	0.61	105.3	14.7	50
80	Polyethersulfone (PES)	GO	Tensile	1	1.16	0.79	31	25	47
81	PES	GO	Tensile	1.3	0.15	0.052	NA	NA	48
82	PES	GO	Tensile	1	0.187	0.031	3.4	0.44	49

[a] GO = graphene oxide, rGO = reduced GO, GOf = functionalized GO.
[b] Tensile = stress strain tests, DMTA = dynamic mechanical thermal analysis; the storage modulus (E') at 30 ºC is considered for calculating enhancement.
[c] When GO is reported in vol.%, conversion to wt.% was performed using polymer and GO densities of 1.3 and 2.0 g/cm^3 respectively.
[d] Reported tensile strength for nanocomposites is the maximum tensile value reported when maximum enhancement in Young's modulus is attained.

Supplementary data file references:

1 Zhao, J., Wang, X., Zhou, W., Zhi, E., Zhang, W., Ji, J. Graphene-reinforced biodegradable poly(ethylene succinate) nanocomposites prepared by in situ polymerization. *J. App. Poly.Sci.* **130**, 3212-3220 (2013).

2 Wan, C., Chen, B. Reinforcement and interphase of polymer/graphene oxide nanocomposites. *J. Mater. Chem.* **22**, 3637-3646 (2012).

3 Sturer, P., Wissert, R., Thomann, R., Mulhaupt, R. Functionalized Graphenes and Thermoplastic Nanocomposites Based upon Expanded Graphite Oxide. *Macromol. Rapid Commun*. **30**, 316-327 (2009).

4 Cao, Y-C. *et al.* A poly (ethylene oxide)/graphene oxide electrolyte membrane for low temperature polymer fuel cells. *J. Power Sources* **196**, 8377-8382 (2011).

5 Lee, D. C., Yang, H. N., Park, S. H., Kim, W. J. Nafion/ graphene oxide composite membranes for low humidifying polymer electrolyte membrane fuel cell. *J. Membr. Sci.* **452**, 20-28 (2014).

6 Li, W. *et al*. A facile method to produce graphene oxide-g-poly (L-lactic acid) as a promising reinforcement for PLLA nanocomposites. *Chem. Engg. J.* **237**, 291-299 (2014).

7 Cai, D., Song, M. A simple route to enhance the interface between graphite oxide nanoplatelets and a semi-crystalline polymer for stress transfer. *Nanotech*. 20, 315708/1- 315708/6 (2009).

8 Achaby, M. E. *et al.* Graphene oxide reinforced chitosan/polyvinylpyrrolidone polymer bio-nanocomposites. *J. Appl. Poly. Sci.* **131**, 41042/1-41042/11 (2014).

9 Bora, C., Bharali, P., Baglari, S., Dolui, S. K., Konwar, B. K. Strong and conductive reduced graphene oxide/polyester resin composite films with improved mechanical strength, thermal stability and its antibacterial activity. *Compos. Sci. Tech.* **87**, 1-7 (2013).
10 Tang Z. *et al.* Incorporation of graphene into polyester/carbon nanofibers composites for better multi-stimuli responsive shape memory performances. *Carbon* **64**, 487-498 (2013).
11 Wang J. *et al.* Polycarbonate toughening with reduced graphene oxide: toward high toughness, strength and notch resistance. *Chem. Engg. J.* **325**, 474-484 (2017).
12 Lee, B. Y, Park, J. Y., Kim, Y. C. Effect of polycarbonate structure and reduction time on graphene oxide dispersion. *Poly. Adv. Tech.* **26**, 1241-1246 (2015).
13 Shen, B., Zhai, W., Tao, M., Lu, D., Zheng, W. Enhanced interfacial interaction between polycarbonate and thermally reduced graphene induced by melt blending. *Compos. Sci. Tech.* **86**, 109-116 (2013).
14 Sellam, C. *et al.* High mechanical reinforcing efficiency of layered poly (vinyl alcohol) – graphene oxide nanocomposites. *Nanocomp.* **1**, 89-95 (2015).
15 Hwang, S-H., Kang, D., Ruoff, R. S., Shin, H. S., Park, Y-B. Poly (vinyl alcohol) reinforced and toughened with poly(dopamine) treated graphene oxide, and its use for humidity sensing. *ACS Nano* **7**, 6739-6747 (2014).
16 Cheng, H. K. *et al.* Poly (vinyl alcohol) nanocomposites filled with poly (vinyl alcohol)-grafted graphene oxide. *ACS Appl. Mater. Inter.* **4**, 2387-2394 (2012).
17 Cano M. *et al.* Improving the mechanical properties of graphene oxide based materials by covalent attachment of polymer chains. *Carbon* **52**, 363-371 (2013).
18 Morimune, S., Nishino, T., Goto, T. Graphene oxide reinforced PVA nanocomposites. 18th International Conference on composite materials, 2011.
19 Xu, Y., Hong, W., Bai, H., Li, C., Shi, G. Strong and ductile poly (vinyl alcohol)/graphene oxide composite films with a layered structure. *Carbon* 47, 3538-3543 (2009).
20 Cheng-an T. *et al.* Mechanical properties of graphene oxide/ polyvinyl alcohol composite film. *Polym. Polym. Compos.*, **25**, 11-16 (2017).
21 Zhou, T. *et al* The preparation of high performance and conductive poly (vinyl alcohol)/graphene nanocomposite via reducing graphite oxide with sodium hydrosulfite. *Compos. Sci. Tech.* **71**, 1266-1270 (2011).
22 Morimune, S., Nishino, T., Goto, T. Poly (vinyl alcohol)/graphene oxide nanocomposites prepared by a simple eco-process. *Polym. J.* **44**, 1056-1063 (2012).
23 Kashyap, S., Pratihar, S. K., Behera, S. K. Strong and ductile graphene oxide reinforced PVA nanocomposites. *J. Alloys Comp.* **684**, 254-260 (2016).
24 Yang, X., Li, L., Shang, S., Tao, X-M. Synthesis and characterization of layer-aligned poly (vinyl alcohol)/graphene nanocomposites. *Polymer* **51**, 3431-3435 (2010).
25 Zhao, X., Zhang, Q., Chen, D. Enhanced Mechanical Properties of Graphene-Based Poly (vinyl alcohol) Composites. *Macromolecules*, **43**, 23578-2363 (2010).
26 Pandele A. M. *et al.* Synthesis, characterization, and in vitro studies of graphene oxide/chitosan–polyvinyl alcohol films. *Carbohydr. Polym.*, **102**, 813-820 (2014).

27 Goncalves, G. Graphene oxide modified with PMMA via ATRP as a reinforcement filler. *J. Mater. Chem.*, **20**, 9927-9934 (2010).

28 Wang, Y., Shi, Z., Yin, J. Kevlar oligomer functionalized graphene for polymer composites. *Polymer* **52**, 3661-3670 (2011).

29 Valles, C., Kinloch, I. A., Young, R. J., Wilson, N. R., Rourke, J. P. Graphene oxide and base-washed graphene oxide as reinforcements in PMMA nanocomposites. *Compos. Sci.Tech.* **88**, 158-164 (2013).

30 Jang, J. Y., Kim, M. S., Jeong, H. M., Shin, C. M. Graphite oxide/poly (methyl methacrylate) nanocomposites prepared by a novel method utilizing macroazoinitiator. *Compos. Sci.Tech*. **69**, 186-191 (2009).

31 Li, Y. *et al.* In situ polymerization and mechanical, thermal properties of polyurethane/graphene oxide/epoxy nanocomposites. *Mater. & Design* **47**, 850-856 (2013).

32 Y-J. Wan *et al.* Lai, Grafting of epoxy chains onto graphene oxide for epoxy composites with improved mechanical and thermal properties. *Carbon* **69**, 467-480 (2014).

33 Li, Z. *et al.* Control of the functionality of graphene oxide for its application in epoxy nanocomposites. *Polymer* **54**, 6437-6446 (2013).

34 Bao C. *et al.* In situ preparation of functionalized graphene oxide/epoxy nanocomposites with effective reinforcements. *J. Mater. Chem.* **21**, 13290-13298 (2011).

35 Gudarzi, M. M., Sharif, F. Enhancement of dispersion and bonding of graphene-polymer through wet transfer of functionalized graphene oxide. *eXpress Polym. Lett.* **6**, 1017-1031 (2012).

36 Li, M., Jeong, Y. G. Poly (ethylene terephthalate)/exfoliated graphite nanocomposites with improved thermal stability, mechanical and electrical properties. *Composites Part A; Appl. Sci. Manuf.* **42**, 560-566 (2011).

37 Yoonessi, M. *et al.* Graphene polyimide nanocomposites; thermal, mechanical, and high-temperature shape memory effects. *ACS Nano* **6**, 7644-7655 (2012).

38 Zhang L-B. *et al.* Preparation, mechanical and thermal properties of functionalized graphene/polyimide nanocomposites. *Compos. Part A: Appl. Sci. Manuf.* **43**, 1537-45 (2012).

39 Cao, L., Sun, Q., Wang, H., Zhang, X., Shi, H. Enhanced stress transfer and thermal properties of polyimide composites with covalent functionalized reduced graphene oxide. *Compos. Part A: Appl. Sci. Manuf.* **68**, 140-48 (2015).

40 Park O-K. *et al.* Synthesis and properties of iodo functionalized graphene oxide/polyimide nanocomposites. *Compos. Part B: Engg.* **56**, 365-371 (2014).

41 Luong, N. D. *et al.* Enhanced mechanical and electrical properties of polyimide film by graphene sheets via in situ polymerization. *Polymer* **52**, 5237-5242 (2011).

42 Kong, J- Y. Preparation and properties of polyimide/graphene oxide nanocomposite films with Mg ion crosslinker. *Euro. Poly. J.* **48**, 1394-1405 (2012).

43 Chen, M. *et al.* Dielectric and mechanical properties and thermal stability of polyimide–graphene oxide composite films. *Thin solid films* **584**, 232-237 (2015).

44 Wang, Y., Shi, Z., Fang, J. Xu, H., Yin, J. Graphene oxide/polybenzimidazole composites fabricated by a solvent-exchange method. *Carbon* **49**, 1199-1207 (2011).

45 Yang, L. *et al.* Design and preparation of graphene/poly(ether ether ketone) composites with excellent electrical conductivity. *J. Mater. Sci.* **49**, 2372-2382 (2014).

46 Zhan, Y. *et al.* Cross-linkable nitrile functionalized graphene oxide/poly (arylene ether nitrile) nanocomposite films with high mechanical strength and thermal stability. *J. Mater. Chem.* **22**, 5602-5608 (2012).

47 Forati, T., Atai, M., Rashidi, A. M., Imani, M., Behnamghader, A. Physical and mechanical properties of graphene oxide/polyethersulfone nanocomposites. *Poly. Adv. Tech.* **25**, 322-328 (2014).

48 Lee, J. H. *et al.* Graphene oxide nanoplatelets composite membrane with hydrophilic and antifouling properties for wastewater treatment. *J. Membr. Sci.* **448**, 223-230 (2013).

49 Ionita, M., Pandele, A. M., Crica, L., Pilan, L. Improving the thermal and mechanical properties of polysulfone by incorporation of graphene oxide. *Compos. Part B: Engg.,* **59**, 133-139 (2014).
